# Von Hippel-Lindau Syndrome: Medical Syndrome or Surgical Syndrome? A Surgical Perspective

**DOI:** 10.15586/jkcvhl.v9i1.206

**Published:** 2021-12-05

**Authors:** Danilo Coco, Silvana Leanza

**Affiliations:** 1 Department of General Surgery, Ospedali Riuniti Marche Nord, Pesaro, Italy;; 2 Department of General Surgery, Carlo Urbani Hospital, Jesi, Italy

**Keywords:** clear cell renal cancer, Pheocromocytoma, PNET, Von Hippel-Lindau

## Abstract

Von Hippel-Lindau syndrome (VHL) is an autosomal dominant disease caused by a genetic aberration of the tumor suppressor gene VHL and characterized by multi-organ tumors. The most common neoplasm is retinal or cerebral hemangioblastoma, although spinal hemangioblastomas, Renal Clear Cell Carcinoma (RCCC), pheochromocytomas (Pheo), paragangliomas, Pancreatic Neuroendocrine Tumors (PNETs), cystadenomas of the epididymis, and tumors of the lymphatic sac can also be found. Neurological complications from retinal or CNS hemangioblastoma and metastases of RCCC are the most common causes of death. There is a strong association between pheochromocytoma and VHL syndrome, and pheochromocytoma is often a classic manifestation of the syndrome. RCCCs are often incidental and identified during other tests. Between 35 and 70% of patients with VHL have pancreatic cysts. These can manifest as simple cysts, serous cysto-adenomas, or PNETs with a risk of malignant degeneration or metastasis of no more than 8%. The objective of this retrospective study is to analyze abdominal manifestations of VHL from a surgical point of view.

## Introduction

In 1894, Collins reported a bilateral incidence of retinal hemangioblastomas in twins, which were referred to as nevi, whereas the German ophthalmologist Von Hippel discussed cases of retinal angiomatosis. In 1926, the Sweden pathologist Lindau identified an association between the retinal angiomatosis and the hemangioblastomas in the cerebellum. In the 1960s, the disease was termed Von Hippel-Lindau disease (VHL) (1–3). From 1960 to 1990, VHL disease was diagnosed on the basis of clinical signs and symptoms. However, in 1988, Seizinger et al. (4) identified an alteration on the short arm of chromosome 3 (3p25–3p26). In 1993, it became possible to isolate the gene responsible for the disease, which was named the VHL gene (4,5). VHL is an autosomal dominant disease caused by genetic aberration of the tumor suppressor gene. It is characterized by multi-organ tumors of the central nervous system, kidney, pancreas, and adrenal and reproductive organs. The most common neoplasm is retinalor cerebral hemangioblastoma, although spinal hemangioblastomas, Renal Clear Cell Carcinoma (RCCC), pheochromocytomas (Pheo), paragangliomas, Pancreatic Neuroendocrine Tumors (PNETs), cystadenomas of the epididymis, and tumors of the lymphatic sac can also be found. Life expectancy is approximately 60 years for men and 50 years for women. VHL related mortality is due to complications from CNS and RCCC tumors (6–8). The onset of symptoms appear in the second decade of life; it therefore develops in childhood or adolescence. It has an incidence rate of 3:100,000 people per year. The VHL gene is located on chromosome 3p25, which consists of three exons for the pVHL gene (a tumor suppressor protein). When the pVHL is not produced due to mutations in the VHL gene (mainly deletions), angiogenesis and uncontrolled cell proliferation are promoted. Therefore, the pVHL mutations seriously increase the risk of tumor growth in the organs indicated (8–10). Approximately 70% of the VHL cases are hemangioblastomas and clear renal cell carcinoma (RCC). The latter is often the main factor affecting prognosis, with kidney cancer being the most common cause of death. For these patients, there is an association between VHL and (i) PNETs with an incidence ranging from 5 to 18% and (ii) (Pheo) that occur in up to 20% of the patients (11–15).The objective of this retrospective study is to analyze abdominal manifestations of VHL from a surgical point of view.

## Materials and Methods

We conducted a retrospective evaluation of PubMed articles. A total of 6537 results appeared when searching for VHL. The pool of results were then massively reduced by only selecting articles from 2000 to 2021. A combination of medical subject heading (MeSH) terms and keywords were used for the search. The inclusion criteria were “Von Hippel-Lindau; Pheocromocytoma; Clear Cell Renal Cancer; PNET.” The primary outcome measure was “VHL surgical treatment.” Two authors analyzed the articles. The methodologies applied in the articles were randomized controlled trials (RCTs), cohort studies, case–control studies, randomized studies, and prospective and retrospective studies. Studies in all languages were included. We excluded case reports and articles not focused on surgical management.

## Results

In a retrospective study, Zwolak et al. (16) found that in the general population, PNETs in VHL have an estimated incidence of approximately 5–18%. However, PNETs comprise only 5% of pancreatic cancers and the association between PNETs and VHL is less than 0.5%. Distant metastases are found in 60–90% of sporadic PNETs, whereas in VHL the incidence of metastases is estimated at 11–20%. Sporadic non-secreting neuroendocrine tumors of the pancreas should be removed when they are greater than 20 mm in diameter. Smaller tumors can be observed if they do not exhibit radiological or histopathological features of malignancy. Hes et al. (17) conducted a genetic analysis of VHL, targeting the formation of pheochromocytomas. They highlighted 3 types of VHL associated with 3 subtypes classification: Type 1, not from pheochromocytomas and mutations in patients with this type of VHL; Type 2, which has the appearance of pheochromocytomas and can be further subdivided into 3 subtypes: low (type 2A), and high (type 2B) risk of RCC, type 2C families with pheochromocytoma only, type 3 Chuvash polycythemia.. In an extensive review of the relevant literature, Varshney et al. (18) found multiple bilateral renal cysts in 50–70% of patients with VHL, and RCC in 30% of patients with VHL. In the third or fourth decade of life, renal cysts are often multiple but asymptomatic and without renal insufficiency or with RCC. They noted that RCC, which is the main cause of mortality in this group of patients, has an incidence of 70% after the age of 60. Pheochromocytomas occur in up to 20% of VHL patients in the second decade of life; these are bilateral or multifocal and secrete catecholamines. The treatment of choice for pheochromocytomas is surgical resection using laparoscopic approaches. Approximately 35–70% of the patients with VHL have pancreatic cysts. Baghai et al. (19), in their retrospective study at Mayo Clinic Hospital, analyzed 109 patients affected by VHL. They found 46% of RCCC, 76% of cerebral or cerebellar hemangioblastoma, 63% of retinal angioma, 16% of pheocromocytoma, 3% of paraganglioma, and 43% of pancreatic lesions. After open or laparoscopic adrenalectomy for Pheo, mean size was between 1.5 and 6.5 cm with no invasive tumors or metastases (16 patients), and 50% of these had concurrent procedure for pancreatic cancer or renal cancer (Table 1).

**Table 1:** Review of studies on VHL.

Author, year	Number of patients	Research type	Retinal or Encephalus Hemangioblastoma	Pheocromocytoma	RCCC	Pancreatic Cancer	Other	Type of VHL	Gene
Zwolak et al. (16)		Retrospective	N/A	N/A	N/A	Incidence: 5–18% distant metastases: 11–20%	N/A		Exon 3 mutation
Hes et al. (17)	N/A	Retrospective		Pheo				VHL 2A,B,C	VHL Type 2: Elongin C by the -domain of pVHL, missense mutations
Varshney et al. (18)	N/A	Retrospective	60% of patients RB, 60–80% HB	up to 20% of VHL, bilateral and occasionally multifocal, 2nd decade of life	Incidence 30%, 70% over 60 years	About 35–70% of patients with VHL have pancreatic cysts. Resection for lesion 3 cm or genetic risk or growth.	–	Type 2 A,B,C for Pheo	–
Baghai et al. (19)	109 patients	Retrospective	83 patients (76%) Cerebellar Hemangioblastoma, 69 patients (63%) Retinal Angioma	17/109(16%) Pheo,3 patients paraganglioma	50 patients (46%)	43 patients (43%)	3 patients (3%) paraganglioma	N/A	N/A

## Discussion

VHL disease is an autosomal, dominant hereditary tumor syndrome with an estimated prevalence of 2–3 per 100,000 people. It predisposes people to hemangioblastoma in the retina, cerebellum, and spine; renal cell carcinoma, pheochromocytoma; islet cell tumors of the pancreas; endolymphatic sac tumors; and cysts in the pancreas and epididymis. Neurological complications from retinal or CNS hemangioblastoma and metastases of RCC are the most common causes of death (5,8,20). The current clinical classification of VHL comprises three types, 1-2-3. Of these, type 2 contains three subtypes. Type 1 is characterized by a high risk of renal cell carcinoma. Types 2a and 2b are characterized by a high risk of both renal cell carcinoma and pheochromocytoma, whereas type 2c is characterized by a high risk of pheochromocytoma. Type 3 is characterized by a risk of Chuvash polycythemia (8–10) (Table 2). Currently, there are no guidelines for the management of hemangioblastoma. Patients with hemangioblastoma inoperable for local dimensions or who are not fit because they are at a high risk of operative complications can be managed with conventional fractional radiotherapy or stereotaxic radiosurgery. Small hemangioblastomas appear to benefit from conservative treatment with radiological follow-up. Therefore, only symptomatic hemangioblastomas should be managed operatively (Weil). Preoperative embolization of hemangioblastomas also appears to be acceptable and safe; this procedure reduces blood loss during surgery and the incidence of postoperative complications (21).There is a strong association between pheochromocytoma and VHL syndrome, and pheochromocytoma is often a classic manifestation of the latter. Pheochromocytoma occurs in up to 20% of patients with VHL in the second decade of life and can be bilateral or multifocal. It typically develops in young adults and its location may be adrenal or extra-adrenal. Often, they may exhibit a low secretory activity while classic symptoms (such as tachycardia, sweating, tachypnea, headache, angina, and palpitations) may be absent. The treatment of choice for pheochromocytomas is surgical resection using either a laparoscopic transperitoneal, extraperitoneal, or robotic laparoscopic approach (22,23).This approach yields excellent results with no recurrence (24,25). RCCs are often incidental and identified during other tests. Partial nephrectomy is recommended for 3 cm tumors. Nephro-sparing on lesions of 3 cm or greater result in 10 year cancer survival rates of up to 81%. Percutaneous and laparoscopic radiofrequency ablation therapy is effective in the treatment of smaller tumors (<3 cm) (26,27). The pancreatic lesions found in VHL disease are a heterogeneous group comprising the following: PNETs, simple cysts, serous cystadenomas, and mixed neuroendocrine neoplasms. Cysts are the most common and have been found in 17–72% of the cases (28–30). Pancreatic resection is performed only for PNETs measuring at least 2 cm in size or any size if they have malignant characteristics. Small tumors can be observed if no radiological or histopathological features of malignancy are exhibited. If a pancreatic NET is small, enucleation is performed. A distal pancreatectomy is used to treat PNETs present in the tail and body of the pancreas. Surgical options involve removing only the tail of the pancreas or performing a pancreatic splenectomy via open surgery or laparoscopy or robotics. A Whipple procedure is used to treat pancreatic NETs found in the head of the pancreas. A central pancreatectomy is used to treat small, low grade tumors (31,32). Blansfield et al. (13) identified three main factors that can determine the risk of metastasis: tumor size, exon 3 mutation, and mean doubling time. There is an apparent 12% association between neuroendocrine tumors of the pancreas and pheochromocytomas in patients with VHL disease (33). Approximately 35–70% of patients with VHL have pancreatic cysts. These can manifest as simple cysts, serous cysto-adenomas, or PNETs with a risk of malignant degeneration or metastasis of no more than 8%. The resection criteria are given by a major tumor of 3 cm or a pathogenic variant in exon 3 and tumor with a growth rate doubling in fewer than 500 days. Because these characteristics increase the risk of metastasis, enucleation, Whipple, or distal pancreatectomy is indicated depending on the site (13,34, 35). In recent years new medical therapies targeting the VHL/HIF axis have been investigated, with some of them being used in clinical trials. The most commonly utilized are tyrosine kinase inhibitors (TKIs), which can decrease angiogenesis by blocking the VEGF pathway. Several TKIs including sunitinib, cabozantinib, axitinib, lenvatinib, and pazopanib are currently being evaluated in phase II clinical trials. These therapies work by inhibiting cancer cell proliferation, metastasis, and the development of therapeutic resistance by suppressing tyrosine kinase receptors (36). Recently, studies were investigated on belzutifan, development code: MK-6482, which is a hypoxia-inducible factor-2α (HIF-2α) inhibitor for the treatment of adult patients with Hippel-Lindau syndrome. This agent is not FDA approved in US and had a 83% response rate in PNETs (37–39).

**Table 2:** Type of VHL, clinical findings, gene mutations, and notes.

Type of VHL	Clinical findings	Gene	Notes
Type 1	Retinal and CNS HB, RCCC, pancreaticcysts, and neuroendocrine tumors	Missense mutations	Decreased risk for Pheo
Type 2			
Type 2A	CNS and retinal HB Pheo	Missense mutation	Low risk for RCC
Type 2B	RCCC, Retinal HB, CNS HB,pancreatic cyst, and neuroendocrinetumors, Pheo	–	High risk of RCC
Type 2C	Pheo	–	High risk of Pheo
Type 3	Chuvash polycythemia.	–	

CNS: central nervous system; HB: hemangioblastoma; RCCC: Renal Clear Cell Carcinoma; Pheo: Pheocromocytoma.

## Conclusions

VHL syndrome exhibits a wide range of signs and symptoms and is often difficult to diagnose. It is a multisystem tumor syndrome with common manifestations affecting the CNS and various visceral organs. VHL patients often require multidisciplinary treatment, including genetic counseling and personnel such as surgeons, radiologists, pathologists, and oncologists. Early identification of mutations leads to early intervention and reduces rates of morbidity and mortality. Although the use of surgery to treat hemangioblastomas yields promising results, RCC appears to benefit considerably from a combination of surgery and the use of targeted therapies such as VEGF. The future promises an even greater understanding of the genetic structure of the disease, facilitating its treatment with increasingly specific drugs.
